# Japanese Patients’ and Physicians’ Preferences for Anticoagulant Use in Atrial Fibrillation: Results from a Discrete-choice Experiment

**DOI:** 10.36469/9904

**Published:** 2015-06-12

**Authors:** Ken Okumura, Hiroshi Inoue, Masahiro Yasaka, Juan Marcos Gonzalez, A. Brett Hauber, Bennett Levitan, Zhong Yuan, Jean Baptiste Briere

**Affiliations:** 1 Division of Cardiology Hirosaki University Graduate School of Medicine, Hirosaki-shi, Aomori, Japan; 2 Second Department of Internal Medicine University of Toyama School of Medicine, Toyama-shi, Toyama, Japan; 3 Department of Cerebrovascular Disease National Kyushu Medical Center, Fukuoka-shi, Fukuoka, Japan; 4 RTI Health Solutions Research Triangle Park, North Carolina, USA; 5 Janssen Research & Development, LLC, Titusville, New Jersey; 6 Global Health Economics & Outcomes Research Department Bayer Pharma AG, Berlin, Germany

**Keywords:** benefit-risk, bleeding, stroke prevention, discrete-choice experiment, conjoint-analysis, anticoagulants, atrial fibrillation, physician preferences, patient preferences

## Abstract

**Background:** Anticoagulants are recommended for stroke prevention in patients with atrial fibrillation (AF), but are associated with an increased risk of bleeding; therefore, physicians face benefit-risk tradeoffs when prescribing anticoagulants to AF patients. Although the unmet medical need for safer anticoagulants has been well-documented, there is limited information about the importance that patients and physicians place on cardiovascular events.

**Objectives:** The aim of this study was to quantify patients’ and physicians’ willingness to accept tradeoffs between the benefits and risks of anticoagulants in order to 1) document the potential differences between patients’ and physicians’ perceptions of benefits and risks and 2) support physicians’ clinical decision making.

**Methods:** Preferences from Japanese AF patients and board-eligible or board-certified physicians were elicited using a discrete-choice experiment. Random-parameters logit models were used to estimate importance weights for treatment-related changes in the annual risk of stroke, myocardial infarction, embolism, and bleeding.

**Results:** Japanese patients (N=152) and physicians (N=164) showed different preferences. In particular, among non-fatal outcomes, patients considered disabling stroke to be 16 times more important than non-major clinically relevant bleeding and 2.6 times more important than extra-cranial major bleeding. In contrast, physicians considered the same stroke risk to be 2.7 times more important than non-major clinically relevant bleeding and equally important as major bleeding.

**Conclusions:** Results suggest that Japanese patients are willing to tolerate a greater risk of bleeding in exchange for stroke prevention than are Japanese physicians. The findings demonstrate the importance of physician-patient communication in treatment decisions involving stroke preventative therapies.

## BACKGROUND

Approximately 830,000 people currently live with atrial fibrillation (AF) in Japan.[1] AF is the most common arrhythmia seen in clinical practice[2] and is associated with substantial morbidity and mortality.[3] It is well known that AF is a predisposing factor to thrombus formation in the atria that eventually may travel through the left ventricle to block blood flow to the brain, leading to stroke. The incidence of stroke has been shown to be as much as five times greater among patients with AF than those without AF.[3] Strokes associated with AF are nearly twice as likely to be fatal, and are often accompanied by functional deficit.[4,5] Patients with cardioembolic strokes are more likely to have difficulty in walking, speaking or swallowing, and require constant nursing care and assistance after discharge as compared to lacunar stroke patients.[6] While rhythm control strategies have been widely used for years to manage heart rhythm and reduce the duration of arrhythmia in AF patients[7,8], anticoagulants remain a pivotal preventive therapy for AF patients.[9]

Anticoagulants are effective in preventing stroke among AF patients; however, these drugs are often associated with risks of bleeding, including risks of major intra- and extra-cranial bleeding, and non-major clinically relevant bleeding.[10-12] Studies have shown that patients for whom anticoagulants are indicated[13] often receive sub-therapeutic levels of treatment or no treatment even when treatment is advised by clinical guidelines.[11,14-18] This sub-therapeutic level of treatment is believed to be the result of concerns about bleeding and the tradeoff between benefits and risks of anticoagulants[14,19]; however, there is limited information about the importance that patients and physicians place on cardiovascular events associated with anticoagulant use.[20]

The aim of this study was to quantify patients' and physicians' willingness to accept tradeoffs between the benefits and risks of anticoagulants in order to 1) document the potential differences between patients' and physicians' perceptions of benefits and risks and 2) support physicians' clinical decision making. To achieve these goals, we used a discrete-choice experiment (DCE) to quantify preferences for annual event rates associated with anticoagulants as perceived by the Japanese AF patients and physicians.

## METHODS

### Survey

Respondents were asked to complete a series of choice questions, each including a pair of hypothetical anticoagulants defined by therapy-induced changes in the annual incidences of cardiovascular events. Choices among hypothetical treatments indicate the extent to which treatment outcomes satisfy patients' needs or wants.[21] The pattern of choices reveals the importance weights for events associated with anticoagulant use.

A set of events and event definitions was developed to capture key endpoints measured in clinical trials of anticoagulants. Clinical experts were consulted to finalize the list and definitions of events. The range of annual incidences for each event was determined by reviewing results of randomized controlled trials. These levels were reviewed with clinical experts to ensure that the ranges used in the survey encompassed those seen in anticoagulant clinical trials and clinical practice. The draft survey instruments were pretested using in-depth, semi-structured interviews with eight patients and nine physicians in the United States, and six patients and four physicians in the United Kingdom. The survey was then translated into Japanese, reviewed by three Japanese clinical experts, and pretested using in-depth, semi-structured interviews with two AF patients and one physician in Japan. The survey questions and event definitions were refined based on the pretest findings to ensure the cultural appropriateness and clarity of the event descriptions, as well as the appropriateness of the cognitive effort required to answer the choice questions. The survey design and implementation was conducted according to the checklist outlined as "good research practices" by the *International Society for Pharmacoeconomics and Outcomes Research (ISPOR)*.[22]

The events and incidences for 1 year over which preferences were evaluated in this study are presented in Table 1. They were also presented in graphical format to aid respondents in understanding risks (Figure 1). All events other than all-cause death were defined as non-fatal. Strokes were defined by severity (disabling or non-disabling) rather than origin (ischemic or hemorrhagic) based on the modified Rankin Scale, which measures the level of disability or dependency in daily activities in those who have suffered a stroke or other neurological disorders.[23] Non-disabling stroke was defined as an event with short-term effects (modified Rankin score 0-2), and disabling stroke was defined as an event with long-term or permanent effects (modified Rankin score 3-5). Non-central nervous system (CNS) systemic embolism and myocardial infarction are also included as they are ischemic events associated with AF.[24,25]

Anticoagulants are also associated with adverse events including extra-cranial major bleeding (typically gastrointestinal bleeds) and non-major clinically relevant bleeding.[26,27] Bleeding events were distinguished using the same bleeding criteria by the International Society on Thrombosis and Haemostasis (ISTH) for major and non-major clinically relevant bleeding as used in many clinical trials.[27-31]

**Table 1.** Events and Incidences Used in Preference Surveys

| Events* | Incidence Rates |
|---|---|
| Non-disabling stroke (minor stroke) | 0% chance |
| | 1% chance |
| | 3% chance |
| Disabling stroke (major stroke that results in permanent disability) | 0% chance |
| | 1% chance |
| | 3% chance |
| Non-CNS, systemic embolism (blood clot in the leg) | 0% chance |
| | 2% chance |
| | 5% chance |
| Myocardial infarction (heart attack) | 0% chance |
| | 4% chance |
| | 9% chance |
| Non-major clinically relevant bleeding (moderate bleeding) | 0% chance |
| | 14% chance |
| | 25% chance |
| Extra-cranial major bleeding (non-fatal major bleeding) | 0% chance |
| | 3% chance |
| | 8% chance |
| All-cause death (death from any reason) | 1% chance |
| | 3% chance |
| | 4% chance |
| | 6% chance |

*Apart from all-cause death, all events presented in this table are non-fatal events.
Names in parentheses correspond to the event name used in the patient survey. CNS: central nervous system

Clinical trials of anticoagulants typically record the incidence of all-cause death as an endpoint.[27-31] Therefore, the survey included a follow-up question to assess the importance of all-cause death relative to the non-fatal events in this study.

Patients were asked to choose therapies for themselves while physicians chose therapies for each of four virtual patients (Table 2). The final survey instrument included four sections: 1) background questions intended to characterize the sample and prescribing patterns among physicians, 2) a description of each event associated with anticoagulant use for one year, 3) a set of choice questions, and 4) an all-cause death follow-up question.

**Table 2.** Virtual Patient Profiles

| Profile Number | Key Characteristics | |
|---|---|---|
| Profile 1 | • 71 year-old female<br>• $CHADS_2$=2<br>• No information on prior stroke<br>• No information on prior MI | • No prior bleeding<br>• Takes medication for hypertension and diabetes<br>• CrCl=65mL/min<br>• Active |
| Profile 2 | • 80 year-old female<br>• $CHADS_2$=4<br>• Prior stroke<br>• No information on prior MI<br>• No information on prior bleeding | • Takes medication for hy  pertension and osteoarthritis<br>• Best blood pressure=160/70mmHg<br>• Fractured hip from fall |
| Profile 3 | • 76 year-old male<br>• $CHADS_2$=3<br>• No prior stroke<br>• Prior MI and mild CHF<br>• No information on prior bleeding | • Takes medication for hypertension and hypercholesterolemia<br>• CrCl=40mL/min<br>• Deteriorated renal function<br>• hypercholesterolemiaCrCl=40mL/min<br>• Deteriorated renal function |
| Profile 4 | • 79 year old male<br>• $CHADS_2$=6<br>• Prior stroke<br>• No information on prior MI<br>• No information on prior bleeding | • Takes medication for hypertension and CHF<br>• CrCl=30mL/min<br>• Homebound |

$CHADS_2$: Score estimating the risk of stroke in AF patients. Higher score means higher risk; AF: atrial fibrillation, CrCl: creatinine clearance, HCT: hydrochlorothiazide, MI: myocardial infarction; CHF: congestive heart failure, TIA: transient ischemic attack

To reduce the difficulty of answering the choice questions, all events but all-cause death were organized into four groups: stroke, myocardial infarction, non-CNS systemic embolism and bleeding. The events within each group were mutually exclusive and represented different severities of the same event. Only one event in each group was shown in each choice question. For example, if the annual incidence of non-disabling stroke was shown in a choice question, respondents were not shown the annual risk of disabling stroke in the same choice question. An example of a physician choice question is presented in Figure 1.

**Figure 1.** Example of a Choice Question in the Physician Survey

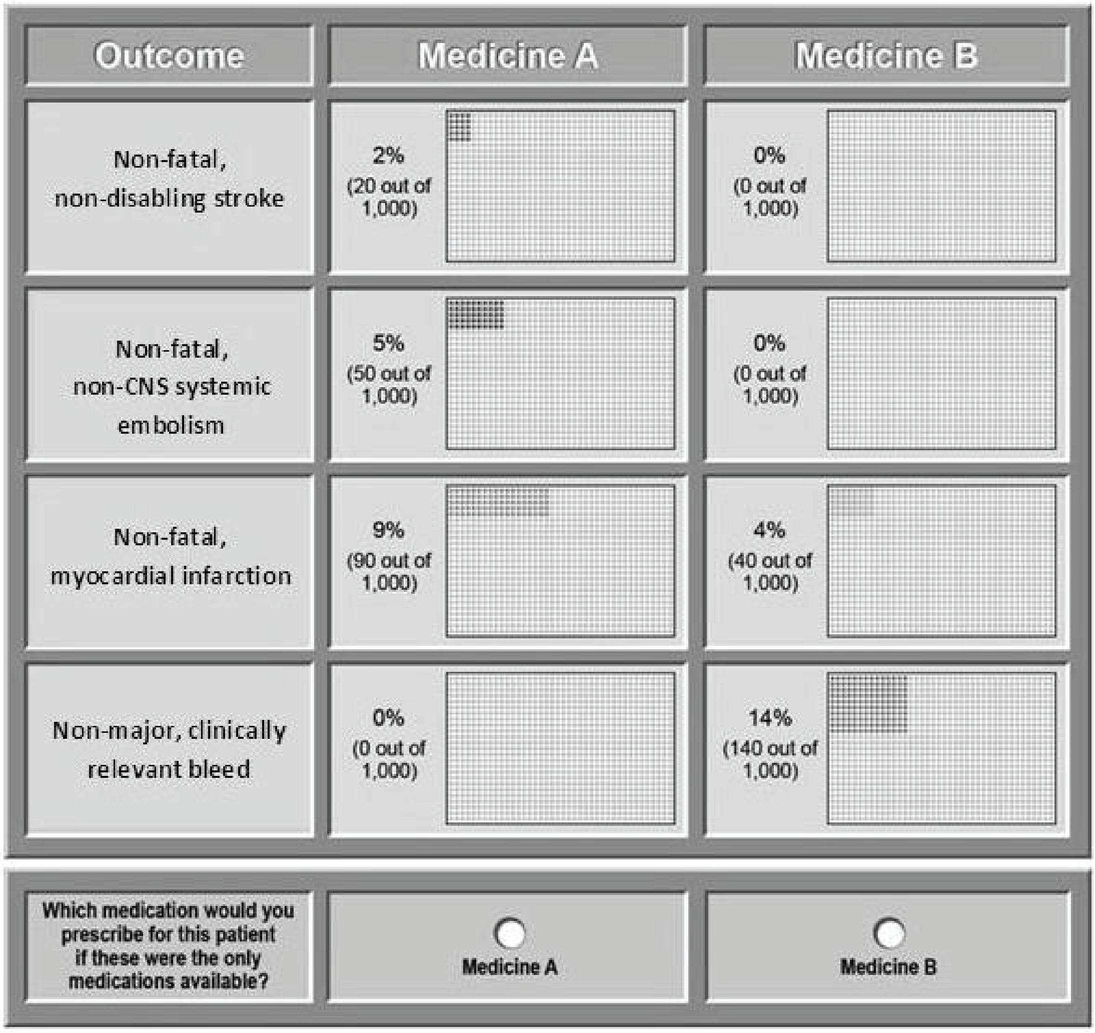

The combinations of annual incidences that define the treatment profiles in each choice question were created using a D-optimal experimental design that resulted in 54 hypothetical treatment pairs. The experimental design was created using our own implementation of a D-optimal algorithm to search for a near-optimal experimental design.[32]

To generate an experimental design that would only include the annual incidence of four outcome measures in each choice question, we imposed overlap restrictions during the optimization of the experimental design. Overlap restrictions required that the annual incidence of specified outcomes was the same across treatment alternatives. Because the levels of these attributes did not differ across alternatives in a choice question, they were assumed to have no impact on treatment choice and therefore were not presented in the choice questions.[33]

To further reduce the cognitive burden of answering all 54 questions in the experimental design, we divided the full set of paired comparisons into six blocks of questions for patients and nine blocks for physicians. Each block had the same number of questions, and questions were not repeated across blocks. Patients answered nine choice questions each. Each physician answered six choice questions for each of the four virtual patients. Thus, each physician answered a total of 24 questions. Patients and physicians were randomly assigned to one of the survey versions. To avoid systematic inattention to a subset of treatment profiles, the order of the choice questions was randomly assigned.

In the follow-up question, respondents were asked to reconsider their response to one of the previous choice questions if the therapy they originally selected was associated with a greater chance of all-cause death than the treatment they rejected.

### Survey Sample

Japanese patients and physicians were recruited from an online consumer panel. Online panels have been increasingly used as sources of subjects in survey research.[34,35,36] Email invites for screening were sent only to the panel members who were considered potentially eligible. Patients who confirmed that they were at least 45 years of age and had a self-reported physician diagnosis of AF were invited to complete the survey, whereas physicians who confirmed that they were board-eligible or board-certified cardiologists, internists, neurologists, or neurosurgeons who currently treat AF patients were invited to complete the survey. The 25-minute online survey was administered between November 2011 and January 2012. Japanese patients who participated in the study received panel points equivalent to approximately US$6, while physicians who participated in the study received an honorarium equivalent to US$50. The survey was approved by RTI International's Office of Research Protection and Ethics (Research Triangle Park, North Carolina). All patients participating in the study provided online informed consent.

### Study Analysis

Random-parameters logit models were used to analyze the choice data. In random-parameters logit models, the dependent variable is the treatment choice and the explanatory variables include the annual incidences of each event included in the choice questions. The model was used to estimate a coefficient (importance weight) for each event incidence per year indicating the relative strength of preference for each annual event incidence. For each importance weight, the model estimates a random parameter to control for the degree of heterogeneity in the importance weights across respondents.[21] Physician and patient data were analyzed separately. All confidence intervals around the importance weights were estimated using a Krisnky-Robb method.[37]

## RESULTS

### Sample

Email invitations to complete the survey were sent to 171 members of an online panel who confirmed having a self-reported physician diagnosis of AF and to 622 physicians who also confirmed that they met the eligibility criteria. Among those invited, 156 patients (90.7%) and 165 physicians (26.5%) completed the survey. Of those who completed the survey, four patients and one physician had no variation in their responses (i.e., always chose the same medication) and were deleted from the sample. The final samples used in the analyses included 152 patients and 164 physicians. Tables 3 and 4 summarize the demographic characteristics of the AF patients and physicians in the final sample, respectively.

**Table 3.** Sample Characteristics: Patients (n = 152)

| Demographic Characteristics | N = 152 |
|---|---|
| Mean age, years (SD) | 63.3 (9.05) |
| Female | 18 (11.8%) |
| College degree or more | 71 (46.7%) |
| **Treatment Experience** | |
| Diagnosed with AF for ≥ 5 years | 95 (62.5%) |
| Treated by cardiologist | 105 (69.1%) |
| Medication | |
| Aspirin | 25 (16.4%) |
| Antiarrhythmic medication | 76 (50.3%) |
| Anticoagulant | 103 (67.8%) |
| Heart-valve replacement surgery | 10 (6.6%) |
| Surgery to remove a clot | 5 (3.3%) |
| **Experience with Cardiovascular Events** | |
| Minor stroke or TIA | 24 (15.9%) |
| Major stroke | 6 (4.0%) |
| Blood clot in the leg | 3 (2.0%) |
| Heart attack | 36 (24.0%) |
| Moderate bleeding not caused by injury | 22 (14.6%) |
| Major bleeding (non-stroke) | 3 (2.0%) |

AF: atrial fibrillation, SD: standard deviation, TIA: transient ischemic attack

**Table 4.** Sample Characteristics: Physicians (n = 164)

| Medical Training and Experience | N = 164 |
|---|---|
| **Specialty** | |
| GP/internal medicine | 64 (39.0%) |
| Cardiology | 58 (35.4%) |
| Neurosurgeon/neurologist | 42 (25.6%) |
| ≥ 10 years of experience | 158 (96.3%) |
| Institution with > 100 beds | 110 (67.1%) |
| Treat ≥ 10 AF patients monthly | 132 (80.5%) |

AF: atrial fibrillation, GP: general practitioner

The mean age of patients in the final sample was 63.3 (SD=9.05). Approximately 88.2% of the patients were male, and nearly half (46.7%) had at least a college degree which is consistent with recent estimates of the percentage of adults with tertiary degrees in Japan.[38] Approximately 63% of all patients reported having been diagnosed with AF for at least 5 years. Nearly one in five (19.9%) patients reported having previously experienced a stroke, or transient ischemic attack. Also, approximately two out of three (67.8%) patients reported taking anticoagulants. Finally, approximately 16% of patients also reported having experienced a bleeding not caused by injury.

Most physicians in the study were internists (39.0%), and cardiologists (35.4%), followed by neurologists (13.4%), and neurosurgeons (12.2%). A large majority of physicians had been in practice for at least 10

years (96.3%) and reported treating at least 10 AF patients per month (80.5%). Most physicians (75.0%) reported prescribing anticoagulants to at least 50% of their AF patients.

## Preference Results

To facilitate the interpretation of the preference results, we show the importance estimates for a change in the annual incidence of each event relative to the importance of a change in the annual risk of all-cause death (Figure 2). The relative importance is the ratio of the importance estimate for each event to the mean importance estimate for all-cause death and can be interpreted as the reciprocal of the number of events that patients or physicians would on average consider equivalent to a single death from any cause. For example, patients would regard three non-disabling strokes as equivalent to a single death when the relative importance of non-disabling stroke is 0.32. Based on this, it is possible to rank the events in order of relative importance (Figure 2). The bars around each relative importance estimate represent the 95% confidence interval of the event relative importance.

**Figure 2.** Patient Importance Weights (n = 152)

Importance weights normalized relative to all-cause death and 95% confidence interval corresponding to a 3-percentage-point change in the incidence of each event. Apart from all-cause death, all events are non-fatal.

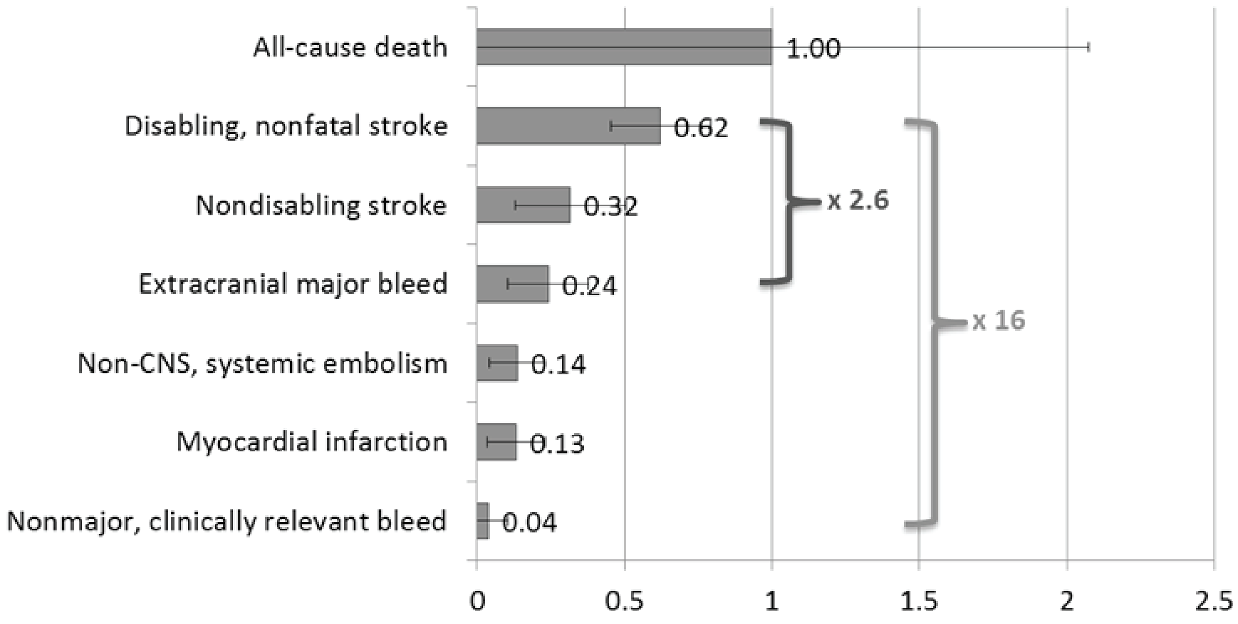

CNS: central nervous system

For patients, all-cause death was the most important event among the events considered in the study. After all-cause-death, patients considered disabling stroke (relative importance of 0.62), non-disabling stroke (relative importance of 0.32), and extra-cranial major bleeding (relative importance of 0.24) to be the most important events associated with treatment. Disabling stroke was approximately 2.6 (0.62 ÷ 0.24) times as important as extra-cranial major bleeding ($p<0.01$). Though not statistically significant, non-disabling stroke was approximately 1.3 (0.32 ÷ 0.24) times as important as extra-cranial major bleeding ($p = 0.88$). Compared with non-major clinically-relevant bleeding (relative importance of 0.04), both disabling and non-disabling strokes were considered far more important by a factor of 16 and 8 ($p<0.01$), respectively.

Interestingly, the importance of myocardial infarction was relatively low (relative importance of 0.13), although 24.0% of patients indicated that they had experienced heart attack prior to this survey.

The ranking of annual events for physicians from most important to least important is shown in Figure 3. As with patients, all-cause death was the most important event for physicians. In general, physicians' relative importance for non-fatal events were lower as compared with those of patients. Especially, physicians considered disabling stroke as far less important compared to death than did patients (relative importance of 0.16 vs. 0.62, respectively). In fact, physicians regarded disabling stroke to be equal in importance to extra-cranial major bleeding (relative importance of 0.16 for both events), about 2.7 times as important as non-major clinically-relevant bleeding (relative importance of 0.06) (0.16 ÷ 0.06) ($p < 0.01$). Although not statistically significant, non-disabling stroke (relative importance of 0.13) was considered less important than extra-cranial major bleeding ($p = 0.29$). The only event that had higher relative importance in physicians than in patients was non-major, clinically relevant bleed, to which patients placed the lowest relative importance. In contrast, the least important event in physicians was myocardial infarction (relative importance of 0.04).

**Figure 3.** Physician Importance Weights (n = 164)

Importance weights normalized relative to all-cause death and 95% confidence interval corresponding to a 3-percentage-point change in the incidence of each event. Apart from all-cause death, all events are non-fatal events.

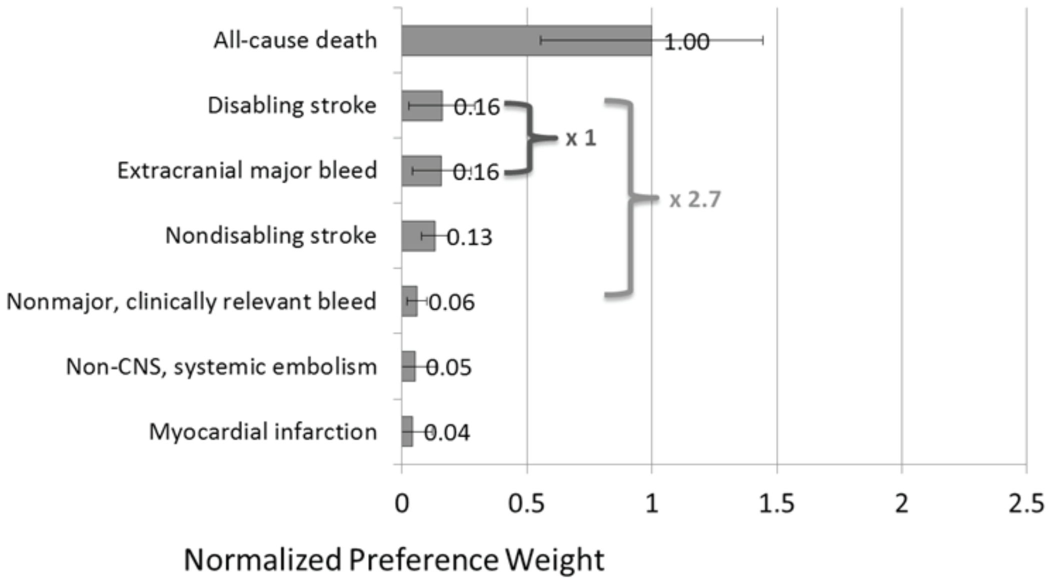

CNS: central nervous system

## DISCUSSION

To our knowledge, this was the first study aimed to quantify the preference of Japanese patients and physicians regarding benefits and risks of anti-coagulant therapy for the prevention of stroke due to AF. The significant impact of AF on stroke-related morbidity and mortality highlights the importance of preventing stroke among AF patients. However, physicians are faced with determining the right balance between benefits and risks of anticoagulants when determining the appropriate approach to stroke prevention. In clinical practice, the decision to use effective stroke-prevention treatments such as warfarin or one of the new generation of anticoagulants is accompanied by the acceptance of an increased risk of bleeding events. This study used a DCE to quantify the benefit-risk tradeoffs that Japanese AF patients and the physicians who treat them are willing to make.

Results from the analysis suggest that Japanese patients and physicians have different preferences for outcomes associated with the use of anticoagulants. In general, physicians considered non-fatal clinical events far less important relative to death, as compared to patients, who were more concerned about the risk of stroke than that of bleeding. In contrast, physicians were far less concerned about stroke than death and were equally concerned about the risk of major bleeding events. Such views on bleeding events among physicians may be associated with the prior research that Japanese AF patients often receive sub-therapeutic levels of prophylactic treatments for stroke. For example, only half of AF patients received oral anticoagulant treatment in a community-based, prospective survey in Fushimi-ku, Kyoto[18] and in an acute-hospital setting across Japan.[16] Additionally, the low intensity of warfarin therapy, especially in patients <70 years old, has been reported in the J-RHYTHM registry.[39] Although one in four patients had experienced myocardial infarction prior to this research, the relative importance for MI was lower than that of non-disabling stroke, extracranial major bleed, or systemic embolism.

The physicians' relative importance scores were much lower than those of patients due to the normalization of relative importance scores based on all-cause death. It implies that all-cause death was very important for physicians so that the importance scores for all other attributes appear to be relatively lower. It is important to note, however, that the conclusions on the relative importance of annual event incidences for patients and physicians remain the same regardless of the type of event chosen for the normalization.

Although our study was not designed to identify differences in preferences based on the specific characteristics of respondents, several factors appeared to be associated with variations in preferences within our sample. For example, we found statistically significant variation in physician preferences by specialty, the hypothetical patient they were asked to treat, and the average number of patients they reported to treat each month. We also found statistically significant variation in preferences by patients' gender, bleeding history (personal and family history), time since diagnosis, and previous use of anticoagulants. These results suggest that future studies may be warranted to further explore the differences in preferences across specific subgroups of patients and physicians.

A prior study that assessed preferences for oral anticoagulants in AF using best-best DCE was in Australia. The results of the Australian study were similar to the patient-preference results in our study. Although the attributes included in the Australian study were not limited to risk-benefit versus tradeoffs, efficacy (risk of stroke) was found to be more important than safety (bleeding risks or availability of antidote).[40] The results of the Australian study and our study cannot be compared directly because the sample of respondents in that study included members of the general population and only a small percentage of respondents had a history of AF. An older study of Canadian patients and physicians utilized face-to-face interviews and a probability trade-off tool to elicit preferences for avoiding stroke and bleeding.[41] The study concluded that patients were more averse to stroke risk than bleeding-averse while physicians were more averse to bleeding risk than stroke risk.

The relevance of balancing benefits and risks of anticoagulants is reflected not only in prescribing decisions among physicians, but in explicit discussions at the regulatory level.[42] The European Medicine Agency's (EMA) Roadmap to 2015 indicates that "the current risk management plan for medicines should be converted into a "benefit/risk management plan[43], and advocates increased transparency of benefit-risk assessments, more use of quantitative methods, and closer attention to patients' values. Additionally, the goals of the recent reauthorization of the Prescription Drug User Fee Act (Pub. L. No. 112-144, 126 Stat. 993 [2012]) in the United States[44] include the development and testing of a benefit-risk framework for use in regulatory decision-making, and specifically indicate including patient perspectives in the regulatory discussions.[45] Although such movements are not as formalized in Japan, the Pharmaceutical and Medical

Devices Agency (PMDA) has published a guideline to facilitate the evaluation and review of benefits and risks of medicines from its development to the post-marketing phase.[46] The use of quantitative methods or reflecting on patients' values are currently out of scope of this guideline, however, our analysis would provide suitable information in reflecting patients' and physician's values and preferences in pharmacotherapy, if such element becomes part of regulatory discussions in Japan.

Our analysis examined clinical benefits and risks of anticoagulation therapy as attributes of tradeoffs. However, there may well be other factors that impact the preferences of anticoagulation therapy in AF among patients and physicians, such as dosing and frequency, need for frequent monitoring and dose adjustment, medication cost, and interactions with food and other medications. In real-life practice, these factors, in addition to clinical benefits and risks of anticoagulants, likely play an important role in determining the treatment of choice from both patients' and physicians' perspectives. In order to better understand their preferences in anticoagulation therapy more comprehensively, further research is warranted in the preference of these factors.

## CONCLUSIONS

The study findings suggest that Japanese patients are willing to tolerate a greater risk of bleeding in exchange for stroke prevention than are Japanese physicians caring for AF. Although treatment decision remains largely the doctor's responsibility, a transparent communication between the physician and the patient is vital in disease management. This study raises an important question about whether and how the patient's perspective should be taken into consideration in stroke-prevention efforts, as our results suggest that Japanese patients' views on risks and benefits of anticoagulant therapies are potentially different from the views of Japanese physicians.

### Limitations

The primary limitation of this study is that the choice questions with which we ask subjects to evaluate anticoagulants, and in the case of physicians, to make prescription recommendations based on the information provided about virtual patients were hypothetical. Differences can arise between stated and actual choices. In the present study, potential hypothetical bias is minimized by offering alternatives that mimic real-world trade-offs as closely as possible.

Secondly, DCEs rely on patients' and physicians' understanding of the treatment features and choice questions. The resulting preference estimates may be influenced by patients' and physicians' interpretation of the event definitions and treatment profiles presented in the survey. In the present study, we attempted to mitigate any potential misinterpretation of the events characterizing anticoagulant treatments by having three experts in the field of AF review the survey and conducting pretest interviews with AF patients and physicians.

Furthermore, some demographic and clinical characteristics of our AF patient sample appeared to differ from those in prior research or registries.[17,18,29,39] For example, the mean age of AF patients were 69.7 years and 71.1 years in the J-RHYTHM registry and J-ROCKET AF trial, respectively, while it was 63.3 years in our study.[29,39] In addition, nearly 90% of our sample was male, as compared to approximately 70% in the J-RHYTHM registry and 80% in the J-ROCKET AF trial.[29,39] These differences indicate that the sample may not be representative of the Japanese AF patients, which limits the generalizability of the survey results.

## ACKNOWLEDGEMENTS

The authors express their gratitude to Dr. Kenji Takeuchi for his assistance during the adaptation of the survey, and Vikram Kilambi and Ryan Ziemiecki for their assistance with data analysis for this study. They would also like to thank Emi Fujinuma for coordinating the communication between authors during the finalization of the manuscript.

**Conflict of Interest Declaration**

This study was funded by Bayer Yakuhin Ltd. KO, HI and MY are paid consultants to Bayer Yakuhin, Ltd. JMG and ABH are employees of RTI Health Solutions, an independent scientific research organization. BL is an employee of Janssen Pharmaceutical Research and Development, and a stockholder in Johnson & Johnson, Baxter International, Inc., Pharmaceutical Holdrs Trust, and Zimmer Holdings, Inc. ZY is also an employee of Janssen Pharmaceutical Research and Development, and a stockholder in Johnson & Johnson. JBB was an employee of Bayer Yakuhin, Ltd. at the time the study was conducted.
